# Primary Shoulder Arthroplasty Versus Conservative Treatment for Comminuted Proximal Humeral Fractures: A Systematic Literature Review

**DOI:** 10.2174/1874325001004020087

**Published:** 2010-02-17

**Authors:** Dennis den Hartog, Jeroen de Haan, Niels W. Schep, Wim E. Tuinebreijer

**Affiliations:** 1 Department of Surgery-Traumatology, Erasmus MC, University Medical Center Rotterdam, ‘s Gravendijkwal 230, 3015 CE Rotterdam, The Netherlands; 2 Department of Surgery and Traumatology, Westfriesgasthuis, Maelsonstraat 3, 1624 NP Hoorn, The Netherlands

**Keywords:** Proximal humeral fractures, arthroplasty, shoulder fractures, review, therapy.

## Abstract

The objective was to identify whether arthroplasty or conservative treatment is the best available treatment for three- and four-part proximal humeral fractures by analyzing the outcome measure of the Constant score. We conducted an electronic search. The systematic review included 33 studies encompassing 1096 patients with three- or four-part proximal humeral fractures that used the Constant score as outcome measure. The mean Constant score in the conservative group was 66.5 and in the arthroplasty group was 55.5. The difference could be attributed to selection bias, unreliable classification of the fractures and inter-observer differences in the assessment of the Constant score.

## INTRODUCTION

Proximal humeral fractures are one of the most frequent osteoporotic fractures in the elderly. In 1993, the age-adjusted incidence in the Finnish population (per 100,000 people aged ≥60) of proximal fractures was 106 for women and 41 for men [1]. The incidence of all humeral fractures, per 10,000 person years, in an Australian population aged ≥ 60 years was 54.8 for women and 22.6 for men [2]. In an United States Medicare population over 65 years of age, proximal humeral fractures accounted for ten percent of the fractures in this age group [3]. Risk factors for these fractures are increasing age, female gender and Caucasian race [3]. Another risk factor is osteoporosis, confirmed by femoral neck bone mineral density [2].

Proximal humeral fractures are classified with the four segment classification by Neer, which is still the most commonly used classification system [4, 5]. The four major segments are the auricular segment (head), the lesser and greater tuberosity and the shaft. These segments are called fragments or parts when they are displaced from the other fragments by 10 mm or 45° of angulation. Treatment is essentially based on this classification. In the majority of cases, these fractures are not displaced and can be treated without surgery. In a prospective consecutive series of 1,027 proximal humeral fractures, only nine percent were three-part and 3% were four-part fractures [6]. Theoretically, in three- and four-part fractures, the blood supply of the humeral head can be jeopardised, which may result in avascular necrosis of the humeral head resulting in a loss of function.

Operative treatment can be defined as closed or open reduction with internal fixation or primary arthroplasty. Only one randomised controlled trial (RCT) of 49 patients has compared conservative treatment with arthroplasty for four-part fractures [7]. This study found better function and less pain in the arthroplasty group. Many reviews about the treatment of proximal humeral fractures have been published. Among these, one quantitative systematic review was found in the literature [8]. In this review, Misra *et al*. used the percentage of good to excellent results for pain and range of motion as outcome measures. In comparing conservative treatment with arthroplasty, they extracted data from one comparative trial, three case series of conservative treatment and a single five case series of arthroplasty. They concluded that conservatively managed patients had more pain and a poorer range of motion than the arthroplasty patients. In a recent review, it was stated, on the basis of 66 retrieved articles, that the evidence from the published literature was low and did not support any specific treatment choice [9]. Another recent review described the Constant score as the most often used functional score as an outcome measure in studies of proximal humeral fractures [10]. The Constant-Murley scoring system is rated from 0 to 100 and combines the patient’s subjective pain score (out of 15 points) and function scores for activities of daily living (20 points) with an objective assessment of range of motion (40 points) and muscle strength (25 points) [11]. The primary objective of this systematic review of the literature was to identify whether arthroplasty or conservative treatment is the best available treatment for three- and four-part proximal humeral fractures by quantitatively analyzing the outcome measure of the Constant score.

## MATERIALS AND METHODOLOGY

We conducted an electronic search including MEDLINE, EMBASE, LILACS and the Cochrane Central Register of Controlled Trials (CENTRAL). We did not limit the search by language or publication date. We used the following search terms in different combinations as MeSH (Medical Subject Heading) terms and as text words: Proximal humeral fracture, arthroplasty, treatment outcome, surgery, controlled clinical trial, and comparative study. Manual searches including reference lists of all included studies were used to identify trials that the electronic search may have failed to identify.

Two reviewers independently extracted the data for the primary and secondary outcomes and entered the data into data collection forms developed for this purpose. Discrepancies were resolved by discussion. All data were entered into PASW Statistics 17.0. The following variables were retrieved from the studies when available: year of publication, sample size of three-part and/or four-part fractures, percentage of three-part fractures, conservative treatment, arthroplasty, female/male ratio, age, Constant score with standard deviation (SD), follow-up in months.

The relationship between the Constant score and treatment was estimated using a multiple regression allowing for fracture type, female/male ratio, age, year of publication and follow-up. The results are presented as regression coefficients and 95% confidence intervals (CI).

To study the relationships between the Constant score and sample size and the Constant score and year of publication, scatter plots (funnel plots) were drawn between these variables.

## RESULTS

The systematic review included 33 studies encompassing 1096 patients with three- or four-part proximal humeral fractures that used the Constant score as an outcome measure [12-43]. Seven observational studies included conservative treatment (n=100) and 27 studies described operative treatment with arthroplasty (n=996). From the seven conservative studies, three studies included only conservatively treated patients [26, 30, 37], one paper included conservative and arthroplasty patients [34] and three studies compared conservative treatment with osteosynthesis [23, 35, 36]. The other papers only studied arthroplasty patients [12-20, 22, 24, 25, 27-29, 31, 32, 39-43].

We only included arthroplasty patients who were treated primarily. Nearly all patients in the included studies were operated within one month. The mean age in years was 69.7 (SD=4.8) and 75.2% were females. The mean follow-up duration was 3.1 years (min 1; max 13). Because some studies divided the patients into three-part and four-part fracture groups the seven conservative treatment studies encompassed 11 patient groups and the 27 arthroplasty studies encompassed 30 patient groups. Studies with three- and four-part fractures (n=17), but with a common Constant score for both fracture types, were assigned as four-part fracture studies in our analysis when 20% of the fractures or less were three-part proximal humeral fractures. Using this definition, 179 of the patients from 10 studies had three-part fractures, 778 from 26 studies had four-part fractures and 139 from 5 studies had either three- or four-part fractures. The mean sample size of the conservative groups was 9.1 patients (min 4; max 19) versus 33.2 for the arthroplasty groups (min 10; max 167).

One comparative study comparing conservative treatment and arthroplasty was found, but this study did not publish standard deviations [34]. Only 12 studies presented the Constant score with standard deviations (SD).

The mean Constant score in the conservative group was 66.5 (SD=14.4) and in the arthroplasty group was 55.5 (SD=11.1). The mean difference was 10.9 (CI=2.5 to 19.4; p=0.013). When weighted by sample size the mean Constant score in the conservative group was 70.1 (SD=11.9), in the arthroplasty group was 54.7 (SD=9.7) and the mean difference was 15.5 (CI=13.0 to 17.9; p<0.001). When weighted by sample size, the mean Constant pain score in the conservative group was 10.8 (SD=1.4), in the arthroplasty group was 11.1 (SD=1.2) and the mean difference was -0.37 (CI=-0.78 to 0.04; p=0.08). When weighted by sample size, the mean activities of daily living Constant score in the conservative group was 16.2 (SD=.94), in the arthroplasty group was 12.5 (SD=1.5) and the mean difference was 3.7 (CI=3.4 to 4.0; p<0.001). When weighted by sample size, the mean range of motion Constant score in the conservative group was 28.4 (SD=1.8), in the arthroplasty group was 18.2 (SD=2.7) and the mean difference was 10.2 (CI=9.6 to 10.8; p<0.001). When weighted by sample size, the mean power Constant score in the conservative group was 14.8 (SD=7.1), in the arthroplasty group was 8.9 (SD=5.0) and the mean difference was 5.9 (CI=3.8 to 8.0; p < 0.001).

When only analyzing the studies, which presented standard deviations of the Constant scores, the mean Constant score weighted by sample size in the conservative group was 65.2 (n=38, SD=11.1), in the arthroplasty group was 53.9 (n=255, SD=8.8) and the mean difference was 11.4 (CI=8.2 to 14.5; p<0.001).

When only analyzing the studies, which presented only four-part fractures, the mean Constant score weighted by sample size in the conservative group was 63.3 (n=24, SD=13.7), in the arthroplasty group was 54.9 (n=258, SD=9.6) and the mean difference was 8.4 (CI=2.5 to 14.3; p<0.01).

Table **1** shows the results of the multiple regressions of the Constant score on the fracture type, female/male ratio, age in years, follow-up in years and treatment. The Constant score was significant lower in the arthroplasty group compared with conservative treatment group after controlling for fracture type, female/male ratio, age, year of publication and follow-up.

### Scatter Plots

In Fig. (**1**), a scatter plot (funnel plot) is drawn between the Constant score and the sample size for the arthroplasty group and for the conservative group. In the arthroplasty group, the distribution looks like a funnel with less variation among the larger sample size findings than among the smaller sample size findings. This is what one would expect if the patients were sampled from a single normal underlying population. In the conservative studies, only small sample size studies were found with a large variation in the Constant score. In the arthroplasty group, the shape of the funnel is not only caused by less variation of the Constant score in the larger sample size studies, but also by a lack of studies with larger sample sizes.

In Fig. (**2**), a scatter plot (funnel plot) is drawn between the Constant score and the year of publication for the arthroplasty group and for the conservative group to look for a possible historical trend. In the arthroplasty group, the older studies have higher Constant scores, which was also found in the multiple regression (correlation Spearman’s rho = -0.41). In the conservative studies, the correlation between the Constant score and year of publication is positive: The older studies have lower Constant scores (Spearman’s rho = 0.41). However, these correlations are weak and the Constant scores are scattered throughout the plot.

## DISCUSSION

Only one RCT compares conservative treatment with arthroplasty in four-part humeral fractures [7]. In this study, the arthroplasty patients had a better range of motion and less pain at follow-up between 18 months and 12 years. This prospective study had some flaws: The method of randomization, the blinding of the assessor to the outcomes and the allocation concealment are not mentioned in the publication and the mean age in the operative group was 65.6 years and in the conservative group 70.1 years. Consequently, this study could be biased by these variables. After a literature search, we compared conservative treatment versus arthroplasty of three- and four-part proximal humeral fractures on the functional outcome Constant-Murley score. The analysis was weighted by sample size and yielded a better (higher) mean Constant score for conservative treatment versus patients managed by arthroplasty after allowing for fracture type, female/male ratio, age, year of publication and time of follow-up. The influence of these variables is as follows: Four-part fractures in comparison with three-part, male patients, older patients and patients with longer follow-up have lower Constant scores. The better Constant score in the conservative group was caused by a better range of motion, more power strength and better activities of daily living, but not by a difference in the pain score. Because patients can get a maximum score of 40 for range of motions as part of the Constant score the influence of a better range of motion on the total score is large. When only analyzing studies which presented Constant scores with standard deviations or only studies with four-part fractures, the difference in Constant scores between the conservative and arthroplasty group remained, but the sample size of the conservative group was small.

Our results are in contradiction with the conclusions of the review of Misra *et al*. who found significantly less pain and a significantly better range of motion for the arthroplasty group of patients [8]. However, they only retrieved 5 case series of arthroplasty patients from a total sample size of 149 patients. In addition, they only retrieved studies prior to 1988. We found, for instance, that the older arthroplasty studies had better Constant scores (Fig. **2**). Neither their conservative or arthroplasty papers were used in our analysis, because of missing Constant scores.

Our results for the arthroplasty group are similar to the systematic review of Kontakis *et al*. who calculated a mean Constant score of 56.6 for the arthroplasty patients on the basis of eight papers featuring 560 patients [10]. We included the same six studies, but did not include the two studies with a relative Constant score (expressed as a percentage of the Constant score of the opposite shoulder) as the outcome measure, because these are higher than the normal Constant score.

Our multiple regression analysis controlled for fracture type, female/male ratio, age, year of publication and time of follow-up, but not all of the possible confounders could be retrieved from the studies. For example, the functional status of the patient at the baseline and his/her associated neurological deficits could not be retrieved and this could be a bias that influenced the outcome. Reconstruction of the tuberosity is an important technical consideration in shoulder hemiarthroplasty [14]. These data were also not retrieved from the literature and the effect of this variable on the outcome could not be studied.

**Fig. (1):**
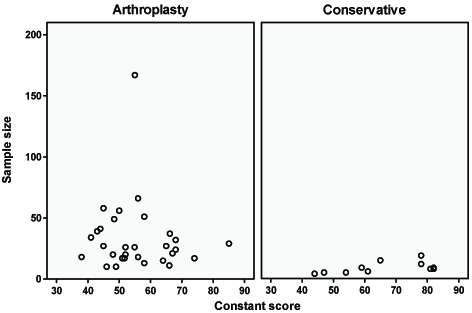
Funnel plot of Constant scores by sample size of the arthroplasty group in 30 patient groups (mean Constant score = 55.5) and of the conservative group in 11 patient groups (mean Constant score = 66.5).

**Fig. (2):**
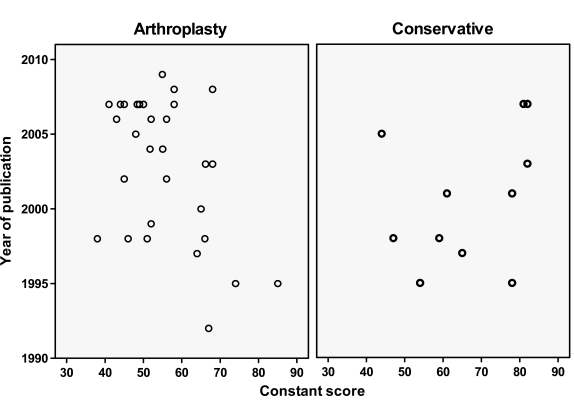
Funnel plot of Constant scores by year of publication of the arthroplasty group in 30 patient groups (mean Constant score = 55.5) and of the conservative group in 11 patient groups (mean Constant score = 66.5).

An important drawback of conservative studies is the small sample sizes in comparison with the arthroplasty studies. This is outlined in the funnel plot (Fig. **1**). This funnel plot was drawn to study publication bias, but small studies with low Constant scores are also published and there are no signs of publication bias. The funnel plot not only shows a lack of conservative studies with large sample sizes, but also too few arthroplasty studies with large sample sizes.

The second funnel plot (Constant score by year of publication) was drawn to study any historical trend in the Constant scores. In psychological reviews it is observed that outcomes are measured more precisely over time and so reduce variation in the funnel plot. This was not seen in this review, but a relationship was found between the Constant score and year of publication depending on the treatment. The recent conservative and arthroplasty studies had higher and lower Constant scores, respectively. This could be caused by the fact that the worst cases are currently not treated conservatively, but by arthroplasty.

A principle problem in studies of proximal humeral fractures is the reliable classification of the fracture types; whether we can reliably diagnose three- and four-part proximal humeral fractures and whether we can measure the Constant score outcomes with validity. The diagnosis and classification of proximal humeral fractures can be difficult and reliability depends on the knowledge of the X-Ray interpreters, the ability of present-day imaging to demonstrate the patho-anatomy of the injury and the anatomical accuracy of the classification [5]. Inter- and intra-observer reliability of the Neer classification is poor [44-46]. The validity of the Constant score has not been studied, but the inter-observer variability in Constant’s original study was an average of 3%, ranging from 0% to 8% [11]. However, the inter-observer differences for the Constant score of two observers were higher in two other studies. In one study, the 95% confidence limits for a single assessment was 16 to 20 points [47] and, in another study, 50% of the differences between two observers were between 10 and 25 points [48]. Conboy *et al*. concluded that such differences make the Constant score insufficiently reliable for clinical follow-up studies [47].

Is the difference in the Constant score in the conservative group versus the arthroplasty patients a real difference or is it the result of bias? In support of a difference, no signs were found of publication bias and the confounding variables had a logical influence on the Constant score. However, evidence against such a difference includes the very small sample sizes of the conservative studies. In addition, the different historical trends in the Constant scores indicate a possible selection bias of patients. The difference could also be attributed to inter-observer differences in the assessment of the Constant score. Prudent conclusions might be that there is no difference between conservative treatment and arthroplasty and that we still do not know which treatment is the best. For this reason, we are planning a RCT comparing conservative treatment with arthroplasty.

## CONCLUSION

In this systematic review we included 33 studies encompassing 1096 patients with three- or four-part proximal humeral fractures that used the Constant score as outcome measure. The mean Constant score in the conservative group was 66.5 and in the arthroplasty group was 55.5. The mean difference was 10.9 (CI=2.5 to 19.4; p=0.013). Using multiple regression analysis the Constant score was significantly lower in the arthroplasty group compared with conservative treatment group after controlling for fracture type, female/male ratio, age, year of publication and follow-up. This difference could be attributed to selection bias (e.g. different prostheses, reconstruction tuberosities), unreliable classification of the fractures and inter-observer differences in the assessment of the Constant score.

## Figures and Tables

**Table 1:** Effects of Fracture Type, Female/Male Ratio, Age, Follow-Up, Year of Publication and Treatment on the Constant Score in 859 Patients

Variable	Coefficient	95% CI	P value	Standardised Coefficient
Fracture type a				
Four-part fracture	-6.1	-7.5; -4.7	<.001	-0.22
Female/male ratio	23.1	16.1; 30.0	<.001	0.25
Age in years	-1.1	-1.3; -0.9	<.001	-0.42
Follow-up in years	-1.2	-1.6; -0.8	<.001	-0.18
Year of publication	-1.4	-1.6; -1.3	<.001	-0.56
Treatment b				
Arthroplasty	-7.4	-9.4; -5.3	<.001	-0.20

a Compared with three-part fracture (reference category).

b Compared with conservative treatment (reference category).
